# Patients undergoing medial patellofemoral ligament reconstruction return to sport sooner and at a higher level than those undergoing concomitant tibial tubercle osteotomy

**DOI:** 10.1002/jeo2.70520

**Published:** 2025-11-28

**Authors:** Michelle E. Kew, William A. Marmor, Elizabeth R. Dennis, Stephanie S. Buza, Theresa Chiaia, Brittany M. Ammerman, Bennett E. Propp, Natalie K. Pahapill, Simone Gruber, Joseph T. Nguyen, Beth E. Shubin Stein

**Affiliations:** ^1^ Hospital for Special Surgery New York New York USA; ^2^ Mount Sinai Doctors New York New York USA

**Keywords:** medial patellofemoral ligament, patellofemoral instability, patient‐reported outcomes, return to sport, tibial tubercle osteotomy

## Abstract

**Purpose:**

The purpose of the study was to compare time to return to sport between patients undergoing isolated medial patellofemoral ligament reconstruction (MPFL‐R) and patients undergoing MPFL‐R and concomitant TTO (MPFL‐TTO) for recurrent patellofemoral instability.

**Methods:**

A retrospective chart review of prospectively collected data was conducted to identify consecutive patients who underwent primary isolated MPFL‐R or MPFL‐TTO. Exclusion criteria included concomitant cruciate ligament surgery, multi‐ligament surgery, failed previous surgery and <1 year follow‐up. Patient demographic information, surgical data, radiographic data, return to sport, and re‐dislocation rates were recorded. Pre‐operative and post‐operative functional outcome scores (Kujala, IKDC), activity level (Pedi‐FABS), and health‐related quality of life (KOOS‐PS, KOOS‐QOL) were compared.

**Results:**

One hundred and seventy‐eight patients were included in the study. One hundred and nineteen patients (74% female) underwent isolated MPFL‐R, and 59 patients (85% female) underwent MPFL‐TTO. Patients who underwent MPFL‐R returned to sport at 9.5 ± 3.9 months, and patients who underwent MPFL‐TTO returned to sport at 12.9 ± 11.0 months (*p* = 0.011). Patients who underwent isolated MPFL‐R had a significantly higher return to the same or higher level of sport compared to MPFL‐TTO (85% vs. 66%, *p* = 0.018). Both cohorts showed significant improvement in KOOS, IKDC, and Kujala scores at all follow‐ups (*p* < 0.001).

**Conclusion:**

Patients who underwent both isolated MPFL‐R and MPFL‐TTO had excellent return to sport rates, with isolated MPFL‐R patients reporting significantly higher rates of return to the same or higher level of sport. The MPFL‐R group returned to sport faster than those who had a concomitant TTO, with no difference in rates of recurrent instability. Patient‐reported outcomes for both groups were significantly improved at 5 years postoperatively. The results of this study contribute to the growing body of literature supporting excellent long‐term functional recovery with low rates of recurrent instability after isolated MPFL‐R or MPFL‐TTO.

**Level of Evidence:**

Level III, cohort study.

**Abbreviations**
CDI: Caton–Deschamps index
KOOS‐PS: Knee Injury and Osteoarthritis Outcome‐Physical function
KOOS‐QOL: Knee Injury and Osteoarthritis Outcome‐Quality of Life
MPFL: medial patellofemoral ligament
MPFL‐R: medial patellofemoral ligament reconstruction
Pedi‐FABS: Pedi‐Functional Activity Brief Scale
PT‐LTR: Patellar Tendon‐Lateral Trochlear Ridge
TDI: Trochlear Depth Index
TT‐TG: tibial tubercle‐to‐trochlear groove

## INTRODUCTION

Acute patellar instability is a disabling injury among young athletes. The estimated incidence is approximately 5.8–43 per 100,000 per year [12], and the risk of recurrent instability is as high as 80% in certain populations [22]. Several anatomic risk factors for dislocation have been identified, including patella alta, trochlear dysplasia, and a lateralized tibial tubercle [7, 12, 29].

Significant debate exists regarding the optimal treatment of recurrent lateral patellar dislocation due to the multifactorial nature of the pathology. Isolated MPFL reconstruction (MPFL‐R) has demonstrated good functional outcomes within the literature [10, 20, 26, 27]. However, failure to address bony malalignment has been proposed as a source of failure for these procedures [26]. The addition of realignment techniques, such as a tibial tubercle osteotomy (TTO), has been recommended to improve patellar stability in select patients with patella alta, increased tibial tubercle‐to‐trochlear groove distance (TT‐TG), trochlear dysplasia, or distal/lateral patellar chondrosis. However, both options are acceptable treatments, and there is no standardised algorithm for the treatment of patellar instability [4, 5]. Prior studies have shown high return to play rates after MPFL reconstruction [10, 16, 20, 22, 27] and MPFL reconstruction with combined TTO (MPFL‐TTO) [21]. Krych et al recently reported favourable functional outcomes and an 85% return to sport rate in a small cohort of patients undergoing isolated MPFL reconstruction and MPFL reconstructions combined with TTO [16]. Additionally, Krych et al. noted that patients who underwent concomitant bony realignment took significantly longer time to return to sport, which was attributed to quadriceps strength deficits noted during functional testing [16]. The majority of patients undergoing surgery to address patellar instability are high school or college age; therefore, return to play timing and decision‐making are paramount in pre‐surgical counselling regarding realistic return to play expectations after these procedures.

The purpose of the current study was to compare time to return to sport between patients undergoing isolated MPFL‐R versus patients who have undergone MPFL‐TTO as treatment for recurrent patellofemoral instability in a large patient cohort. Subjective patient‐reported outcomes of isolated MPFL reconstruction and MPFL with concomitant bony procedures were also compared. It was hypothesised that patients who underwent isolated MPFL reconstruction would return to sport sooner than patients who underwent combined MPFL reconstruction and TTO.

## METHODS

A retrospective chart review of prospectively collected data from 2014 to 2017 was conducted to identify athletes with surgically treated recurrent patellar instability after approval by our institution's Institutional Review Board. This patient cohort was selected as our registry was started in 2014, and the senior author began a multi‐centre study with different inclusion criteria in 2017; therefore, the years of inclusion for this study are limited to 2014–2017. The registry remains active and continues to enrol patients. Patients identified in the database were then confirmed for inclusion in the study through chart review. Patients were included in the study if they underwent primary isolated MPFL reconstruction or primary MPFL reconstruction with concurrent TTO. Patients were indicated for MPFL reconstruction for recurrent patellar instability or first‐time patellar instability with intra‐articular loose body. Patients were indicated for concomitant TTO for recurrent patellar instability with elevated TTTG > 20 or lateral patellar facet chondral lesions. Patients with revision surgery for patellar instability, concomitant cruciate ligament surgery, multi‐ligament surgery, failed previous surgery, and <1 year of follow‐up were also excluded. Patient demographic information, surgical data, radiographic data, and re‐dislocation rates were obtained through chart review. Pre‐operative and post‐operative functional outcome scores (Kujala [17], International Knee Documentation Committee score [IKDC] [1]), activity level (Pedi‐Functional Activity Brief Scale [Pedi‐FABS] [30]), and health‐related quality of life (Knee Injury and Osteoarthritis Outcome‐Physical function [KOOS‐PS], Knee Injury and Osteoarthritis Outcome‐Quality of Life [KOOS‐QOL] [25]) were compared. Pedi‐FABS was included as this scale is used in the principal investigator′s large multi‐centre trial [28]. Pedi‐FABS has been validated for use in adults; therefore, it can be applied to our entire patient cohort. Patients were contacted as part of a standard protocol for inclusion in the database. Each patient was asked about their ability to return to sports, the level of sport, their current level of activity, time to return to sport, and injuries to the operative limb. If a patient did not return to sport, the patient was asked to describe the reasons for not returning.

### Imaging evaluation

Radiographic and magnetic resonance imaging (MRI) evaluation was performed for each patient, and measurements were performed. Tibial tubercle‐to‐trochlear groove distance was reported in groups with measurements >15 mm and >20 mm [8]. Caton–Deschamps index (CDI) was reported in groups with measurements of >1.3, >1.2 and <0.8 [8]. Patellar Tendon‐Lateral Trochlear Ridge (PT‐LTR) was reported as values > 5.5 [24]. Trochlear Depth Index (TDI) was reported as an absolute measurement and as patients meeting criteria for trochlear dysplasia (TDI <3) [3]. MRI images were also evaluated for the presence of chondral lesions and patients with Outerbridge grade III/IV lesions [28] were noted.

### *Surgical technique and rehabilitation* [9]

Patients were placed supine, and regional anaesthesia (spinal) was utilised in all cases. A diagnostic knee arthroscopy was then performed to evaluate the patellar and trochlear chondral surfaces, remove loose bodies, and inspect the knee.

Patients indicated for TTO underwent the following procedure. An incision lateral to the edge of the tibial tubercle was made. The length and width of the osteotomy were planned and marked based on the identified pathology and imaging measurements. The osteotomy was marked with two 0.045 mm K wires, angled in the desired anteroposterior plane. The osteotomy cut was then made with a saw and completed carefully with osteotomes hinged distally in greenstick fashion. The osteotomy was shifted according to the desired correction and secured with two 4.5 mm full‐threaded screws in lag fashion.

A semitendinosus autograft was used for 88% of patients undergoing isolated MPFL reconstruction per surgeon's preference. Semitendinosus allograft was used for 12% of patients undergoing isolated MPFL reconstruction and 100% of patients undergoing MPFL with TTO per surgeon's preference. A 2‐cm incision was made approximately 1&amp;#x02009;cm off the medial edge of the patella, beginning at the level of the superior pole of the patella. The patella was prepared for anchor placement and two anchors were placed, separated by 8–10&amp;#x02009;mm of bone. The graft was then secured to the patella with these anchors. Medial bony landmarks were palpated and sharply exposed. A guide pin was placed in the sulcus, graft isometry was checked and fluoroscopy was used to confirm pin placement. The guide pin was over‐reamed and the graft passed between layers 2 and 3. The graft ends were whipstitched together and secured to the femur using a tenodesis screw. Patellar mobility was examined to ensure that the graft was not tight and that there was a firm endpoint to lateral translation. Knee range of motion was checked to ensure that full flexion and extension were achieved. ﻿

Cartilage restoration procedures were performed if patellar and/or trochlear chondral lesions were noted on preoperative magnetic resonance imaging. Postoperative rehabilitation protocols were not changed with the addition of a cartilage restoration procedure.

Patients undergoing isolated MPFL reconstruction were placed into a hinged knee brace locked in extension for ambulation for 6 weeks. Patients were permitted to bear weight as tolerated and to perform a range of motion exercises without limitations beginning the first day after surgery. The brace was removed at 6 weeks if the patient could perform a straight‐leg raise without a lag. Patients undergoing concomitant TTO were non‐weightbearing for 4–6 weeks with crutches and range of motion exercises from 0° to 90° for 2 weeks, with progression to as tolerated starting at 2 weeks. The senior author prefers non‐weightbearing due to published fracture risk with immediate weight bearing [31]. Weight bearing was progressed gradually between weeks 4 and 6. Postoperative rehabilitation followed our institution′s published rehabilitation protocol. Return to sport clearance was determined by return to sport testing and objective strength and movement data obtained at 6 months postoperatively. Patients were cleared by the primary surgeon after review of return to sport data and physical examination.

### Statistical analysis

Independent samples *t*‐tests were used to compare continuous variables between groups. Categorical variables were assessed using chi‐square and Fisher′s exact tests. Longitudinal changes in patient‐reported outcome measures were analysed using linear mixed‐effects models with random intercepts for each patient to account for within‐subject correlation, while time and procedure type were included as fixed factors. This modelling approach accommodates missing data at intermediate timepoints without requiring imputation, allowing all available observations to contribute to the analysis. The number of patients contributing data at each follow‐up timepoint is reported in the results tables. To account for any potential Type II error from multiple comparisons, a Bonferroni adjustment was applied to all p‐values from the linear mixed models. Critical alpha was set to 0.05 and statistical significance was defined as *p*‐values of 0.05 or below. All analyses were performed using SPSS version 23.0 (IBM Corp., Armonk, NY) and RStudio version 2024.12.0 + 467 (R Foundation for Statistical Computing, Vienna, Austria).

## RESULTS

A total of 178 patients who underwent MPFL‐R or MPFL‐TTO were included in the study (Table 1). 119 patients (74% female) underwent isolated MPFL reconstruction at a mean age of 20.4 ± 7.4 years. 59 patients (85% female) underwent concomitant MPFL reconstruction and TTO at a mean age of 21.4 ± 7.4 years (Table 1). Average follow‐up was 3.5 ± 1.5 years in patients with isolated MPFL‐R and was 3.1 ± 1.5 years in patients undergoing MPFL‐TTO (*p* = 0.131). Patients undergoing isolated MPFL reconstruction had an average of 9.8 ± 19.6 prior dislocation events compared to 12.9 ± 16.1 in the concomitant TTO group (*p* = 0.303). Patients undergoing MPFL‐TTO had significantly higher TT‐TG (19.1 ± 4.1 vs. 14.7 ± 5.2, *p* < 0.001) and PT‐LTR measurements (11.8 ± 7.4 vs. 5.8 ± 6.2, *p* < 0.001) as well as higher rates of grade III/IV patellar chondral lesions (88% vs. 71%, *p* = 0.014). Additionally, patients undergoing MPFL‐TTO had significantly higher rates of concomitant procedures, as listed in Table 1. There was no difference in recurrent instability rates post‐operatively between groups.

**Table 1:** Patient and surgical characteristics.

Group	MPFL (*n* = 119)	MPFL + TTO (*n* = 59)	*p* value
Age	20.3 ± 6.9	23.7 ± 7.9	0.004*
BMI	23.4 ± 4.2	26.5 ± 5.6	<0.001*
Female sex	88 (74%)	50 (85%)	0.128
Time to last follow‐up, years	3.5 ± 1.5	3.1 ± 1.5	0.131
Symptom duration, years	5.4 ± 6.6	9.4 ± 7.5	<0.001*
Number of prior instability events	9.83 ± 19.6	12.9 ± 16.1	0.303
<10	84 (71%)	22 (37%)	<0.001*
≥10	35 (29%)	37 (63%)	
Tourniquet time (min)	47.5 ± 13.6	80.0 ± 15.9	<0.001*
TT‐TG	14.7 ± 5.2	19.1 ± 4.1	<0.001*
TT‐TG > 15 mm	62 (53%)	50 (85%)	<0.001*
TT‐TG > 20 mm	16 (14%)	24 (41%)	<0.001*
CDI	1.14 ± 0.16	1.16 ± 0.23	0.667
CDI ≥ 1.3	19 (16%)	16 (27%)	0.087
CDI ≥ 1.2	37 (31%)	23 (39%)	0.329
CDI ≤ 0.8	1 (1%)	1 (2%)	1.000
Patellar grade III/IV cartilage lesion	85 (71%)	52 (88%)	0.014*
PTI (%)	46.8 ± 16.2	45.8 ± 17.7	0.687
TDI (mm)	2.4 ± 1.4	2.2 ± 1.8	0.421
Trochlear dysplasia (TDI < 3)	73 (61%)	44 (75%)	0.100
PT‐LTR	5.8 ± 6.2	11.8 ± 7.4	<0.001*
PT‐LTR > 5.5	55 (46%)	45 (76%)	<0.001*
TT‐LTR	−8.4 ± 5.9	−4.9 ± 4.4	<0.001*
Concomitant procedure			
PJAC	6 (5%)	20 (34%)	<0.001*
Lat release	13 (11%)	15 (25%)	0.016*
OCA	2 (2%)	6 (10%)	0.017*
Loose body removal	10 (8%)	12 (20%)	0.030*
Graft type			
Autograft	105 (88%)	0	<0.001*
Allograft	14 (12%)	59 (100%)	

Abbreviations: CDI, Caton‐Deschamps Index; MPFL, medial patellofemoral ligament; OCA, Osteochondral Allograft; PJAC, Particulated juvenile allograft cartilage; PTI, Patellartrochlear Index; PT‐LTR, Patellar Tendon‐Lateral Trochlear Ridge Distance; TT‐LTR, Tibial Tubercle‐Lateral Trochlear Ridge Distance; TTO, tibial tubercle osteotomy; TTTG, tibial tubercle to trochlear groove distance.

* Denotes significance.

Both groups had similar rates of pre‐surgical sports participation and level of sport (Table 2). Patients who underwent isolated MPFL‐R returned to sports at an average of 9.5 ± 3.9 months after surgery compared to 12.9 ± 11.0 months in the combined procedure group (*p* = 0.011). The isolated MPFL group had a significantly higher return to the same or higher level of sport (85% vs. 66%, *p* = 0.018). Reasons for not returning to sports are listed in Table 2. There was no difference in return to sport rates between male and female patients, with no difference in return to sport level of play (Table 3).

**Table 2:** Recurrent instability and return to sport.

Group	MPFL		MPFL + TTO		*p* value
	Total	*N* (%)	Total	*N* (%)	
Recurrent instability					
None	114	107 (94%)	55	51 (93%)	0.464
Subluxation	114	2 (2%)	55	3 (6%)	
Dislocation	114	4 (4%)	55	1 (2%)	
Subluxation + dislocation	114	1 (<1%)	55	0 (0%)	
Sports before surgery					
No	118	17 (14%)	59	15 (25%)	0.097
Yes	118	101 (86%)	59	44 (75%)	
Competition level					
Recreational	101	66 (65%)	44	30 (68%)	0.740
Elite^a^	101	35 (35%)	44	14 (32%)	
Returned to sport					
No	101	10 (11%)	44	5 (11%)	0.627
Yes	101	85 (84%)	44	38 (86%)	
Unknown	101	6 (6%)	44	1 (2%)	
RTS same or higher level					
No	85	13 (15%)	38	13 (34%)	0.018*
Yes	85	72 (85%)	38	25 (66%)	
Time to RTS (months), mean ± SD	85	9.5 ± 3.9	38	12.9 ± 11.0	0.011*
Reason for not returning					
Pain	10	1 (10%)	5	1 (20%)	
Instability	10	0 (0%)	5	0 (0%)	
Lack of interest	10	3 (30%)	5	1 (20%)	
Anxiety about knee	10	2 (20%)	5	1 (20%)	
Surgeon recommendation	10	0 (0%)	5	1 (20%)	
Weakness	10	1 (10%)	5	0 (0%)	
COVID‐19 policy	10	0 (0%)	5	1 (20%)	
Contralateral knee surgery	10	1 (10%)	5	0 (0%)	
Unspecified	10	2 (20%)	5	0 (0%)	

Abbreviations: COVID‐19, coronavirus disease‐2019; MPFL, medial patellofemoral ligament; RTS, return to sport; TTO, tibial tubercle osteotomy.

^a^ Elite athletes are defined as athletes who participate in high school, collegiate, professional, national and international competitions.

* Denotes statistical significance (*p* ≤ 0.05).

**Table 3:** Sex differences in return to sport.

Group	Female		Male		*p* value
	Total	*N* (%)	Total	*N* (%)	
Sports before surgery					
No	137	31 (23%)	40	1 (3%)	0.002*
Yes	137	106 (77%)	40	39 (98%)	
Competition level					
Recreational	106	73 (69%)	39	23 (59%)	0.264
Elite^a^	106	33 (31%)	39	16 (41%)	
Returned to sport					
No	106	10 (9%)	39	5 (13%)	0.138
Yes	106	93 (88%)	39	30 (77%)	
Unknown	106	3 (3%)	39	4 (10%)	
RTS same or higher level					
No	93	20 (22%)	30	6 (20%)	0.861
Yes	93	73 (79%)	30	24 (80%)	
Time to RTS (months), mean ± SD	93	11.6 ± 8.8	30	9.2 ± 3.9	0.160

Abbreviations: COVID‐19, coronavirus disease‐2019; MPFL, medial patellofemoral ligament; RTS, return to sport; TTO, tibial tubercle osteotomy.

^a^ Elite athletes are defined as athletes who participate in high school, collegiate, professional, national, and international competitions.

* Denotes statistical significance (*p* ≤ 0.05).

Both groups reported significant improvement in patient‐reported outcome scores (KOOS‐QOL, IKDC, KOOS‐PS, and Kujala) across the study time period, with the exception of the Pedi‐FABS score, which remained stable over time (Supporting Information: Table 1). The isolated MPFL‐R group had significantly higher PROMs at 1‐year and 5‐year time points compared to those undergoing a combined procedure (Supporting Information: Table 1). At the 2‐year time point, the isolated MPFL‐R group had higher PROMs; however, this was not statistically significant (Supporting Information: Table 1).

## DISCUSSION

The main finding in this study was that patients who underwent isolated MPFL reconstruction return to the same or higher level of sports at a significantly higher rate compared to those who underwent MPFL reconstruction with concomitant TTO. The MPFL‐R group returned to sport an average of 4 months sooner than those undergoing concomitant TTO. There was no sex difference noted in return to sport rates or level of play. Both groups demonstrated significant improvement in PROMs from baseline at all post‐operative timepoints. Patients who underwent an isolated MPFL had significantly greater improvement compared to those who underwent an MPFL‐TTO at the 1‐year and 5‐year time‐points.

Return to sport rates after surgery for patellar instability surgery are high and recent systematic reviews evaluate return to sport after isolated MPFL reconstruction [10, 20, 22, 27] and MPFL reconstruction with concomitant bony procedures [6] Manjunath et al. reviewed 20 studies and found an overall return to sport rate of 85.1% for patients undergoing isolated MPFL reconstruction, with 68.3% of patients able to return to a same or higher level of sport [22]. Our study noted an 85% return to the same or higher level of sports for patients undergoing isolated MPFL reconstruction; however, the athletes in our study returned at a later time point than those in Manjunath et al. (9.48 months vs. 7 months). Variability in return to sports rates is difficult to quantify and several studies have reported a lack of objective criteria in return to sport guidelines after patellar instability surgery [18, 33]. The patients in our study returned to sport later than those in prior studies, with Krych et al. [16] reporting return to play at 7 months and a meta‐analysis by Zaman et al. [33] reporting a range of return to sport timelines from 3 months to 6 months postoperatively. Krych et al. [16] reported on objective functional testing after isolated MFPL reconstruction and noted a longer return to play timeline in patients who failed to achieve 85% strength to the contralateral leg. This suggests that a longer return to play timeline, as noted in this study, may allow for athletes to regain strength and functional abilities, thus allowing them to successfully return to sport. Our study builds on the data presented by Krych et al. [16] and reports on a larger cohort, with return to play data and reason for not returning to play. Studies by Chatterji et al. [6] and Zaman et al. [33] highlight the need for objective measurements in return to sport after patellar instability surgery. Of the 92 studies included in both systematic reviews, Chatterji et al. [6] noted that only 16 studies had specific return to play criteria after MPFL‐TTO and Zaman et al. [33] noted 10 studies with objective return to play protocols after isolated MPFL reconstruction. This suggests that objective return to sport criteria is critical for the successful return to play after patellar instability procedures and standardising rehabilitation protocols with respect to brace use, weightbearing, and return to play protocols for patellar instability surgery with and without bony realignment is needed. Future studies should focus on the development of objective criteria evaluating strength and functional abilities to allow for safe return to play. The senior author′s multi‐centre study has captured pilot data to begin establishing a return to sport protocol and guidelines after surgery for patellar instability.

The patients in this study who underwent concomitant TTO showed a lower rate of return to the same or higher level of sport of 66% with a longer return to sport time of 13 months, compared to those undergoing isolated MPFL reconstruction. Krych et al. followed 39 patients after surgery for patellar instability and similarly found a longer return to sport timeline in patients undergoing a concomitant bony procedure (9.8 vs. 7 months) [15] Krych et al. did not comment on the level of return to play, however, patients in the current study undergoing MPFL with concomitant TTO showed a significantly lower return to the same or higher level of play [16]. Feller et al. also found lower return to sport rates in patients undergoing a combined patellar stabilisation procedure [11, 32]. There is no accepted algorithm for deciding between an isolated MPFL and MPFL with TTO to treat patellar instability. Return to sport timing and level is a critical component of a pre‐surgical discussion with a patient and surgeons should be aware of the return to sport differences between these procedures and be thoughtful when deciding to include a TTO.

Several etiologies may be the root cause of lower return to high‐level sports after TTO. In the current study, patients undergoing concomitant bony procedures had a higher rate of grade III/IV patellar chondral lesions and a higher rate of concomitant procedures (chondral procedure, loose body removal, lateral release). This cohort of patients also had a higher rate of prior instability events and lower pre‐surgical patient‐reported outcome scores, suggesting that patients undergoing concomitant bony procedures likely have sustained more damage to the patellofemoral joint and had more functional limitations from their symptoms. This cohort of patients underwent several concomitant surgical procedures to address their long‐standing patellofemoral pathology, which could affect recovery time and lead to lower return to sport rates. Additionally, this patient cohort may have an increased kinesiophobia due to the high rate of presurgical instability events and longer symptom duration. Patients undergoing TTO also have a delayed start to functional rehabilitation, as physical therapy is limited while the osteotomy heals. The delay in rehabilitation will inherently increase the recovery time compared to an isolated soft tissue procedure. Return to sport protocols should be individualised for each procedure, as soft tissue and bony procedures lead to different rehabilitation protocols and timeline to progress to sport.

A critical component of the return to play evaluation is patient readiness for return to sport. The most common reasons for not returning to sport in this study were lack of interest and anxiety about the knee. Hurley et al. followed a cohort of 35 patients who were not able to return to sport after isolated MPFL reconstruction and noted that 25.7% of patients reported ‘lifestyle factors’ as a reason for not returning to sport [14]. Similarly, Manjunath et al. noted that 28.6% of athletes who did not return to play cited lifestyle change as the primary reason [22]. Adolescent patients may not choose to return to sport due to change in schools, loss of interest, or new non‐athletic interests, suggesting that surgery may be used as a means to end a young athletic career to focus on other pursuits. Careful discussion of the patient′s future athletic goals and realistic expectations should be included as a part of preoperative counselling. Fear of reinjury is a common reason for not returning to sport after anterior cruciate ligament reconstruction [2] and outcome measures have been developed to assess an athlete′s readiness to return to sport. Lampros et al. [19] provide an overview of these outcome measures in the setting of patellar instability and recommend uthe se of the anterior cruciate return to sport after injury (ACL‐RSI) measure and the Tampa Scale of Kinesiophobia (TSK‐11) in a return to play protocol after patellar instability surgery [33].

### Limitations

This study has several limitations. First, a retrospective analysis of prospectively collected data was performed, introducing selection bias. The cohort of patients comes from a single‐surgeon database, so results may not be generalisable to the general public. Additionally, the primary surgeon uses primarily autograft in isolated MPFL reconstruction procedures as it is a faster healing graft and allograft for combined bony procedures for patellar instability, as the underlying bony abnormalities are being corrected. Current literature does not show a difference in outcomes or recurrent instability rates with allograft or autograft [13, 23] and further large‐scale studies, such as JUPITER, will allow for further evaluation of outcomes. Finally, patients undergoing concomitant procedures, in addition to TTO, were included, which introduces variability in the analysis. However, as there is no published algorithm for the treatment of patellar instability, our sample is representative of the varied procedures required to appropriately treat patellar instability and provides insight into functional return after these procedures. Additionally, literature comparing outcomes of Bankart repair versus Latarjet has established a precedent for comparisons of outcomes between soft tissue and bony procedures [15].

## CONCLUSION

Patients who underwent both isolated MPFL‐R and MPFLR‐TTO had excellent return to sport rates, with isolated MPFL‐R patients reporting significantly higher rates of return to the same or higher level of sport. The MPFL‐R group returned to sport faster than those who had a concomitant TTO, with no difference in rates of recurrent instability. Patient‐reported outcomes for both groups were significantly improved at 5 years postoperatively. The results of this study contribute to the growing body of literature supporting excellent long‐term functional recovery with low rates of recurrent instability after isolated MPFL‐R or MPFL‐TTO.

## AUTHOR CONTRIBUTIONS

**Michelle E. Kew**: Data analysis, data interpretation, manuscript preparation and editing, and final manuscript approval. **William Marmor**: Data collection, manuscript preparation and editing. **Elizabeth Dennis**: Data collection, data interpretation and final manuscript approval. **Stephanie Buza**: Data collection, data interpretation and final manuscript approval. **Theresa Chiaia**: Data collection, data interpretation and final manuscript approval. **Brittany Ammerman**: Data collection, data interpretation and final manuscript approval. **Bennett Propp**: Data collection. **Natalie Pahapill**: Manuscript preparation and editing, and final manuscript approval. **Simone Gruber**: Data collection, data interpretation and final manuscript approval. **Joseph Nguyen**: Data analysis. **Beth Shubin Stein**: Data interpretation and final manuscript approval.

## CONFLICT OF INTEREST STATEMENT

Beth E. Shubin Stein has received research support from CONMED and AOSSM/OREF, and is a consultant/speaker for Arthrex.

## ETHICS STATEMENT

Hospital for Special Surgery, 2014‐123.

## Supporting information

Supplemental Table 1.

## Data Availability

The data that support the findings of this study are available on request from the corresponding author. The data are not publicly available due to privacy or ethical restrictions.
