# Supplementary material for: Patients undergoing medial patellofemoral ligament reconstruction return to sport sooner and at a higher level than those undergoing concomitant tibial tubercle osteotomy
Source: J Exp Orthop. 2025 Nov 28;12(4):e70520. doi: 10.1002/jeo2.70520 (PMC12661213; doi:10.1002/jeo2.70520)
Supplement: Supplementary file 1 — Supplemental Table 1. [file JEO2-12-e70520-s001.docx]

**Supplemental Table 1: Patient Reported Outcome Measures**

|  |  | **MPFL** | | **MPFL+TTO** | | **Mean** |  | |  |
| --- | --- | --- | --- | --- | --- | --- | --- | --- | --- |
| **PROM** | **Time** | **N** | **Mean [95% CI]** | **N** | **Mean [95% CI]** | **Diff. [95% CI]** | | **P-value [B]** | |
| KOOS QOL | Baseline | 87 | 30.7 [26.2, 35.3] | 46 | 17.5 [11.2, 23.8] | 13.2 [5.4, 21.0] | | 0.001* | |
|  | 1Y | 77 | 70.9 [66.1, 75.8] | 28 | 55.4 [47.3, 63.5] | 15.6 [6.1, 25.0] | | 0.001* | |
|  | 2Y | 82 | 74.0 [69.3, 78.7] | 32 | 61.3 [53.8, 68.9] | 12.7 [3.8, 21.6] | | 0.005* | |
|  | 3Y | 39 | 72.1 65.3, 79.0] | 4 | 54.7 [33.3, 76.1] | 17.4 [-5.1, 39.9] | | 0.129 | |
|  | 4Y | 38 | 75.0 [68.0, 82.0] | 6 | 61.5 [44.0, 79.0] | 13.5 [-5.3, 32.4] | | 0.159 | |
|  | 5Y+ | 32 | 76.2 [68.6, 83.7] | 11 | 54.5 [41.6, 67.5] | 21.6 [6.7, 36.6] | | 0.005* | |
|  | P-value [W] |  | <0.001* |  | <0.001* |  | |  | |
|  |  |  |  |  |  |  | |  | |
| Pedi-FABS | Baseline | 89 | 13.1 [11.3, 14.9] | 46 | 8.8 [6.3, 11.3] | 4.3 [1.2, 7.4] | | 0.006* | |
|  | 1Y | 77 | 12.9 [11.0, 14.9] | 30 | 6.3 [3.2, 9.4] | 6.6 [3.0, 10.3] | | <0.001* | |
|  | 2Y | 82 | 13.7 [11.8, 15.5] | 32 | 8.3 [5.2, 11.3] | 5.4 [1.9, 8.9] | | 0.003* | |
|  | 3Y | 39 | 14.1 [11.4, 16.8] | 4 | 5.8 [-2.7, 14.2] | 8.3 [-0.6, 17.2] | | 0.067 | |
|  | 4Y | 38 | 14.5 [11.8, 17.3] | 6 | 9.0 [2.1, 15.9] | 5.5 [-1.9, 13.0] | | 0.147 | |
|  | 5Y+ | 32 | 13.2 [10.2, 16.2] | 11 | 6.0 [0.9, 11.1] | 7.2 [1.3, 13.2] | | 0.017* | |
|  | P-value [W] |  | 0.933 |  | 0.798 |  | |  | |
|  |  |  |  |  |  |  | |  | |
| IKDC | Baseline | 119 | 48.5 [45.6, 51.4] | 58 | 41.4 [37.2, 45.5] | 7.1 [2.0, 12.2] | | 0.006* | |
|  | 1Y | 78 | 82.1 [78.5, 85.7] | 30 | 69.8 [64.0, 75.6] | 12.3 [5.5, 19.2] | | <0.001* | |
|  | 2Y | 83 | 81.9 [78.4, 85.4] | 32 | 75.9 [70.2, 81.5] | 6.0 [-0.6, 12.7] | | 0.073 | |
|  | 3Y | 39 | 83.1 [78.0, 88.2] | 5 | 66.3 [52.1, 80.5] | 16.8 [1.7, 31.9] | | 0.029* | |
|  | 4Y | 39 | 84.6 [79.5, 89.7] | 6 | 80.0 [67.1, 93.0] | 4.5 [-9.4, 18.5] | | 0.525 | |
|  | 5Y+ | 32 | 82.4 [76.7, 88.0] | 11 | 63.4 [53.9, 73.0] | 18.9 [7.8, 30.0] | | 0.001* | |
|  | P-value [W] |  | <0.001* |  | <0.001* |  | |  | |
|  |  |  |  |  |  |  | |  | |
| KOOS-PS | Baseline | 87 | 33.4 [30.6, 36.3] | 45 | 37.5 [33.5, 41.5] | -4.1 [-9.0, -.8] | | 0.102 | |
|  | 1Y | 76 | 11.8 [8.7, 14.8] | 28 | 17.6 [12.6, 22.7] | -5.8 [-11.7, 0.1] | | 0.052 | |
|  | 2Y | 82 | 11.3 [8.3, 14.2] | 32 | 15.2 [10.5, 19.9] | -4.0 [-9.5, 1.6] | | 0.164 | |
|  | 3Y | 39 | 11.1 [6.9, 15.4] | 4 | 18.2 [4.8, 31.5] | -7.0 [-12.0, 7.0] | | 0.325 | |
|  | 4Y | 38 | 11.1 [6.7, 15.4] | 6 | 8.7 [-2.2, 19.5] | 2.4 [-9.3, 14.2] | | 0.686 | |
|  | 5Y+ | 32 | 11.0 [6.3, 15.7] | 11 | 25.8 [17.8, 33.8] | -14.8 [-24.1, -5.4] | | 0.002* | |
|  | P-value [W] |  | <0.001* |  | <0.001* |  | |  | |
|  |  |  |  |  |  |  | |  | |
| Kujala | Baseline | 118 | 58.1 [55.5, 60.8] | 54 | 48.2 [44.2, 52.1] | 10.0 [5.0, 14.7] | | <0.001* | |
|  | 1Y | 78 | 90.5 [87.3, 93.8] | 30 | 79.8 [74.5, 85.1] | 10.7 [4.6, 16.9] | | 0.001* | |
|  | 2Y | 82 | 89.0 [85.9, 92.2] | 32 | 87.1 [82.0, 92.2] | 1.9 [-4.1, 7.9] | | 0.530 | |
|  | 3Y | 39 | 89.9 [85.3, 94.6] | 4 | 74.5 [60.1, 88.9] | 15.4 [0.3, 30.6] | | 0.045* | |
|  | 4Y | 38 | 91.1 [86.3, 95.7] | 6 | 89.0 [77.2, 100.8] | 2.1 [-10.6, 14.7] | | 0.747 | |
|  | 5Y+ | 32 | 89.8 [84.7, 94.9] | 11 | 75.1 [66.4, 83.8] | 14.7 [4.7, 24.8] | | 0.004* | |
|  | P-value [W] |  | <0.001* |  | <0.001* |  | |  | |

PROM: Patient Reported Outcome Measure

MPFL: Medial patellofemoral ligament

TTO: Tibial tubercle osteotomy

KOOS-QOL: Knee Injury and Osteoarthritis Outcome-Quality of Life

KOOS-PS: Knee Injury and Osteoarthritis Outcome-Physical function

IKDC: International Knee Documentation Committee score

Pedi-FABS: Pedi- Functional Activity Brief Scale

P-value [W]: Within-group change over time for each surgical group, assessed using a linear mixed-effects model.

P-value [B]: Between-group comparison (MPFL vs. MPFL+TTO) at each individual time point, based on estimated marginal means from the linear mixed-effects model.

* denotes statistical significance (P≤0.05)
